# An *Agrobacterium*-Mediated Transient Expression Method for Functional Assay of Genes Promoting Disease in Monocots

**DOI:** 10.3390/ijms24087636

**Published:** 2023-04-21

**Authors:** Haijiao Xu, Qingle Chang, Luli Huang, Peiyao Wei, Yulu Song, Zejian Guo, You-Liang Peng, Jun Fan

**Affiliations:** MOA Key Lab of Pest Monitoring and Green Management, College of Plant Protection, China Agricultural University, Beijing 100193, China

**Keywords:** *Agrobacteria*-mediated transient expression, gain-of-function, functional screening, susceptibility, chloroplast, protein-protein interaction, rice blast disease, barley, *Arabidopsis*

## Abstract

*Agrobacterium*-mediated transient expression (AMTE) has been widely used for high-throughput assays of gene function in diverse plant species. However, its application in monocots is still limited due to low expression efficiency. Here, by using histochemical staining and a quantitative fluorescence assay of β-glucuronidase (GUS) gene expression, we investigated factors affecting the efficiency of AMTE on intact barley plants. We found prominent variation in GUS expression levels across diverse vectors commonly used for stable transformation and that the vector pCBEP produced the highest expression. Additionally, concurrent treatments of plants with one day of high humidity and two days of darkness following agro-infiltration also significantly increased GUS expression efficiency. We thus established an optimized method for efficient AMTE on barley and further demonstrated its efficiency on wheat and rice plants. We showed that this approach could produce enough proteins suitable for split-luciferase assays of protein-protein interactions on barley leaves. Moreover, we incorporated the AMTE protocol into the functional dissection of a complex biological process such as plant disease. Based on our previous research, we used the pCBEP vector to construct a full-length cDNA library of genes upregulated during the early stage of rice blast disease. A subsequent screen of the library by AMTE identified 15 candidate genes (out of ~2000 clones) promoting blast disease on barley plants. Four identified genes encode chloroplast-related proteins: OsNYC3, OsNUDX21, OsMRS2-9, and OsAk2. These genes were induced during rice blast disease; however, constitutive overexpression of these genes conferred enhanced disease susceptibility to *Colletotrichum higginsianum* in *Arabidopsis*. These observations highlight the power of the optimized AMTE approach on monocots as an effective tool for facilitating functional assays of genes mediating complex processes such as plant-microbe interactions.

## 1. Introduction

Transient gene expression is the temporary expression of genes after nucleic acid has been delivered into plant cells [1]. It provides a convenient alternative to stable transformation and has been widely used as an efficient tool for plant biological studies, such as rapid and economical production of recombinant proteins [2], analysis of protein subcellular localization, and protein–protein interactions [3], examination of promoter transactivation and nucleotide binding capacity of transcription factors [4], and functional analysis of genes involved in plant–microbe interactions [5]. Multiple technologies have been used for introducing nucleic acids encoding an expression cassette into plant cells, e.g., the transformation of protoplasts [6,7], particle bombardment [8], virus infection [2], and *Agrobacterium*-mediated transient transformation [3]. Among these, *Agrobacterium*-mediated transient expression (AMTE) has significant advantages for broad deployment because of its simplicity, low cost, rapid and robust expression, and high throughput [9].

The AMTE has been successfully used in a number of plant species [3]. However, major technical obstacles still remain for intact monocot plants such as rice, maize, and barley. Early studies have shown that monocots lack diffusible chemical signals inducing T-DNA circularization or *vir* gene expression in *Agrobacterium* [10]; moreover, metabolites inhibiting the growth of *Agrobacterium* and activating *vir* genes in Ti-plasmid have been uncovered in maize and rice plants [11,12]. These factors may reduce the compatibility of monocots with *Agrobacteria* and suppress T-DNA transfer efficiency. The current scenario for transient functional assay of rice or maize genes mainly involves the utility of the protoplast system [7,13], but not in intact tissues, or leans on heterologous expression in tobacco leaves [14]. Gene expression in protoplasts does not fully replicate the in vivo conditions of intact plants, and traits observed from the transient expression on dicot plants do not always correlate with those from monocots [5,15]. Similarly, for barley plants, transient gene expression frequently relies on particle bombardment [8,16], virus infection [17,18], or protoplast transformation [19], whereas the use of *Agrobacterium*-mediated transformation is still limited due to its low expression efficiency.

Furthermore, plant disease is a complex process involving plant gene reprograming, which mediates multiple signaling and metabolic pathways. To screen for candidate genes potentially involved in the disease process, we have constructed a full-length cDNA library of genes induced in the rice-blast fungus interaction with subtractive hybridization and obtained several library clones capable of inducing cell death through a screen with a transient expression on *Nicotiana benthamiana* plants. However, among these clones, only a few were able to promote the disease caused by blast fungus, while the others showed no impact on the disease’s development, presumably due to the low efficiency of transient expression on barley plants [5]. Therefore, further screening for genes responsible for the disease process requires integrating high-efficiency transient expression with the plant-*Magnaporthe oryzae* interaction.

In this study, we investigated significant factors affecting the levels of AMTE in barley and established a procedure with substantially improved expression efficiency. Subsequently, we screened the library [5] for cDNA clones enhancing the development of blast disease on barley plants. Out of the 2000 individual clones screened, 15 clones harboring genes with diverse functions were identified, four of which encode chloroplast-related proteins. Further characterization of these four genes showed that they were upregulated during fungal infection, and their stable overexpression in *Arabidopsis* transgenic lines strongly enhanced plant susceptibility to *Colletotrichum higginsianum*. Collectively, our work has established an efficient AMTE system in planta, which will significantly facilitate the functional analysis of candidate genes in monocots.

## 2. Results

### 2.1. Binary Vectors Differed in Transient Expression Efficiency of the β-Glucuronidase Gene in Barley

**Figure 1:**
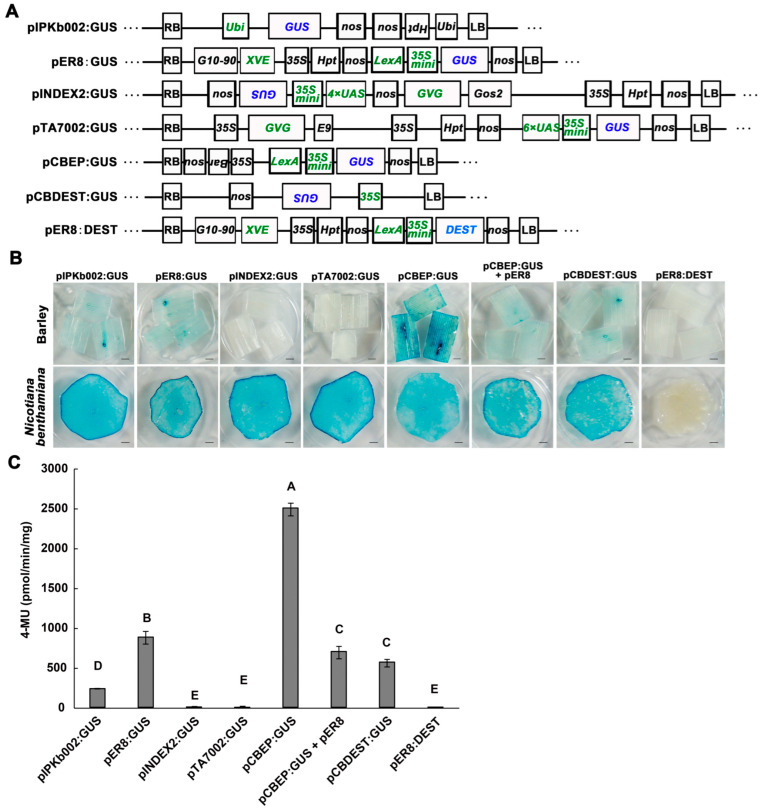
Comparison of transient expression efficiency between different vectors in barley (var. E9) leaves. (**A**). Schematic drawings of binary vectors used in this report. Diverse cis-regulatory elements and promoters in T-DNA were selected for GUS (β-glucuronidase) expression. The empty vector pER8:DEST was used as a negative control. Green letters represent different promoter elements regulating *GUS* transcription: *35S* indicates cauliflower mosaic virus (CaMV) 35S promoter; *Ubi* indicates ubiquitin promoter; *XVE*, an estradiol-activated chimeric transcription activator, controlled by constitutive promoter *G10–90* and targets the LexA operator (*LexA*); *35S mini* indicates −46 to +1 region of the 35S minimal promoter; *GVG*, a dexamethasone (DEX) activated chimeric transcription activator, target *UAS* operator; 4×UAS and 6×UAS indicate four or six copies of GAL4 UAS; *Gos2* indicates the constitutive promoter of rice *GOS2* gene; *nos* indicates terminator; *Hpt* and *Bar* indicate Hygromycin and Basta resistance genes, respectively; RB and LB indicate T-DNA right and left border regions; inverted letters represent the gene orientation (from right to left). (**B**). GUS staining after transient expression with different vectors in barley (**upper**) and *Nicotiana benthamiana* (**lower**) leaves. AGL1 and individual vectors (0.5 OD_600_) were syringe infiltrated into the first leaves of barley following 1 d of moisturizing treatment, then collected for staining analysis at 4 dpi. *N. benthamiana* leaves were infiltrated at the same time as a control. For pER8:GUS, pCBEP:GUS+pER8, and pER8:DEST, 100 μM estradiol was infiltrated 2 h after bacterial infiltration; for pINDEX2:GUS and pTA7002:GUS, 20 μM DEX was infiltrated. Scale bars = 1.5 mm. (**C**). Quantitative fluorescence analysis of GUS activity in barley leaves infiltrated with AGL1 (GUS). Leaf samples were treated and collected as in (**B**). Data shown are mean ± SD (*n* = 6). Different letters indicate significant differences at *p* < 0.01. Three biological replicates were performed.

Previous studies have shown that cis-regulatory elements promote AMTE in *N. benthamiana* [20]. We thus constructed a panel of binary vectors, which carry diverse cis-regulatory elements and promoters in T-DNA driving the expression of the β-glucuronidase gene (GUS) (Figure 1A), to investigate the GUS expression efficiency in barley plants. We transformed *Agrobacterium tumefaciens* strain AGL1 with individual vectors and infiltrated the agro-transformants (0.5 OD_600_) into the leaves of *N. benthamiana* and barley variety E9 plants to analyze the gene expression efficiency by GUS staining and quantitative fluorescence assay. GUS staining of the agro-infiltrated leaf tissues showed that the gene was well expressed for all tested vectors in *N. benthamiana*; however, the staining was less intensive and varied significantly in barley leaves. For vectors pINDEX2:GUS and pTA7002:GUS, in which GUS expression is driven by the upstream activating sequence (UAS) and 35S minimal promoter [21,22], no staining was observed compared with the empty vector control. GUS expressions, driven by a ubiquitin gene promoter (pIPKb002) [23], an inducible promoter (pER8) [24], and the 35S promoter (pCBDEST) [5], were only weakly stained. In contrast, much stronger staining was observed for the pCBEP vector [25], in which the expression was driven by a module consisting of a LexA-binding sequence and a 35S minimal promoter (Figure 1B). Since pER8:GUS and pCBEP:GUS bear the same promoter module driving GUS expression, we speculated that additional components encoded by the T-DNA in pER8 might suppress GUS expression. Indeed, a weakened staining of GUS was observed when we co-inoculated *Agrobacteria* carrying pCBEP:GUS with those carrying pER8 into barley leaves (Figure 1A,B). The variation in GUS expression by diverse vectors was corroborated by measuring the activity of the GUS enzyme in protein extracts from agro-infiltrated barley leaves. The highest level of GUS fluorescence was observed for pCBEP:GUS, more than twice as high as those of pER8:GUS and pCBDEST:GUS. No GUS activity was observed in the negative control (pER8:DEST), pINDEX2:GUS, or pTA7002:GUS (Figure 1C). Further, to confirm that the staining and fluorescence were not from the *agrobacteria*, we deleted the right border of pCBEP:GUS for transient expression. After agro-infiltration, no GUS staining was observed, indicating the GUS expression was dependent on the transfer of T-DNA (Figure S1). We thus selected pCBEP:GUS for further optimization of the transient expression procedure.

### 2.2. Optimization for Higher Efficiency of Transient Expression in Barley Leaves

**Figure 2:**
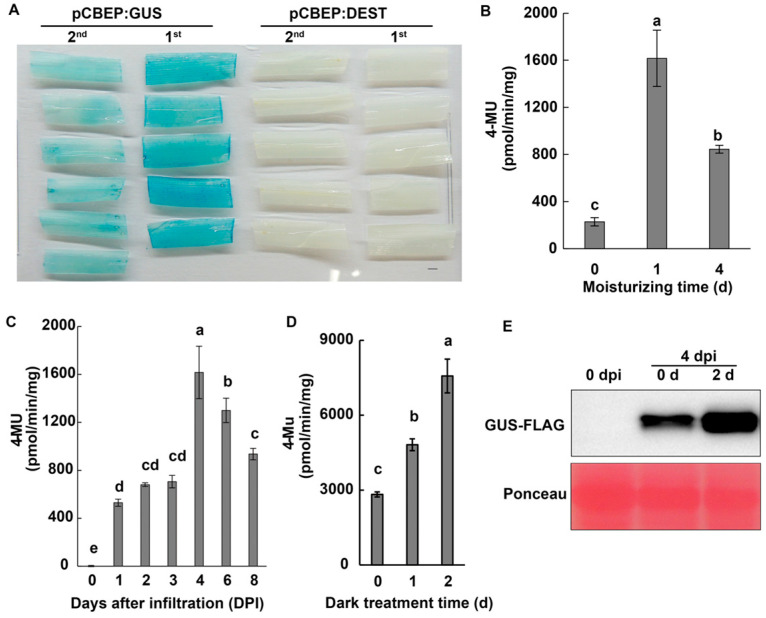
Optimizing conditions for transient expression in barley (var. E9) leaves. (**A**). GUS staining in the first (1st) and the second (2nd) leaves following the infiltration of AGL1 (pCBEP:GUS) at 4 dpi. Empty vector (pCBEP:DEST) used as a negative control. Scale bars = 1.5 mm. (**B**). Quantifying GUS activity under different moisturizing times (0, 1 or 4 d) following the agro-infiltration in the first leaf of barley at 4 dpi. (**C**). Quantifying GUS activity at different cultivation times (0–8 dpi) with 1 d moisturizing after agro-infiltration. (**D**). Quantifying GUS activity under 1 or 2 days of dark treatment before collecting samples at 4 dpi. (**E**). Western blot analysis of FLAG-tagged GUS protein accumulation under dark treatments at 0 dpi (the first line) and 4 dpi (last two lines) with 0 d or 2 d dark treatment. Signals were detected with an anti-FLAG antibody, quantitated, and normalized to the Ponceau loading control. The experiments were syringe-infiltrated with 0.5 OD_600_ of AGL1 (pCBEP:GUS). Data shown are mean ± SD (*n* = 6). Different letters indicate significant differences at *p* < 0.01. Three biological replicates were performed.

Previous studies have shown that multiple factors, including *Agrobacterium* strains, plant seedling stage, and incubation conditions, may contribute to higher levels of transient expression in plants [26,27]. We tested four *Agrobacterium* strains harboring pCBEP:GUS and observed higher levels of GUS activity in barley leaves infiltrated with AGL1 or C58C1 strains than GV3101 or EHA105 strains (Figure S2A), with an optimum bacterial density of OD_600_ 0.5 for AGL1 (Figure S2B). We subsequently used the AGL1 (pCBEP:GUS) strain to infiltrate newly expanded leaves of 1- and 2-leaf-stage barley seedlings. The results showed that the GUS staining was more intense in the first leaves than in the second leaves (Figure 2A); however, similar levels of GUS activity were observed when *Agrobacteria* were vacuum infiltrated or hand-infiltrated with a syringe into the leaves (Figure S2C) or when infiltration buffer with or without acetosyringone (AS) was used (Figure S2D).

In order to test whether high relative humidity could increase the transient expression efficiency, we placed the infiltrated barley plants under a moisturized condition (above 98% relative humidity) for 0, 1, and 4 d before collecting samples at 4 days post-infiltration (dpi). We measured the GUS activity, and results showed that 1 d of moisturizing treatment strongly increased the GUS activity by over 7 folds compared to the control (0 d); however, the effect was attenuated by half in an extended period of high humidity (4 d) (Figure 2B). Using the improved procedure with 1 d of moisturizing treatment, we evaluated the GUS activity in barley leaves during the 8 days following the infiltration of the AGL1 (pCBEP:GUS). The results showed that a low level of GUS activity could be detected at 1 dpi, and it nearly tripled at 4 dpi before gradually decreasing by about 20% and 42% at 6 and 8 dpi, respectively (Figure 2C), indicating that 4 dpi was an ideal time point for evaluation of a transient expression in barley plants.

In addition, it has been reported that the dark treatment of plant seedlings greatly improves the efficiency of AMTE [3,26]. Hence, we integrated the dark treatment into our procedure and found that 2 days of darkness treatment following infiltration of AGL1 (pCBEP:GUS) could increase the GUS activity considerably (Figure 2D). The effect of dark treatment on GUS expression was further confirmed by a protein gel blot assay. A massively enhanced accumulation of the FLAG-tagged GUS protein (size around 72 KDa) could be observed in plants treated with darkness compared with the control (Figure 2E).

**Figure 3:**
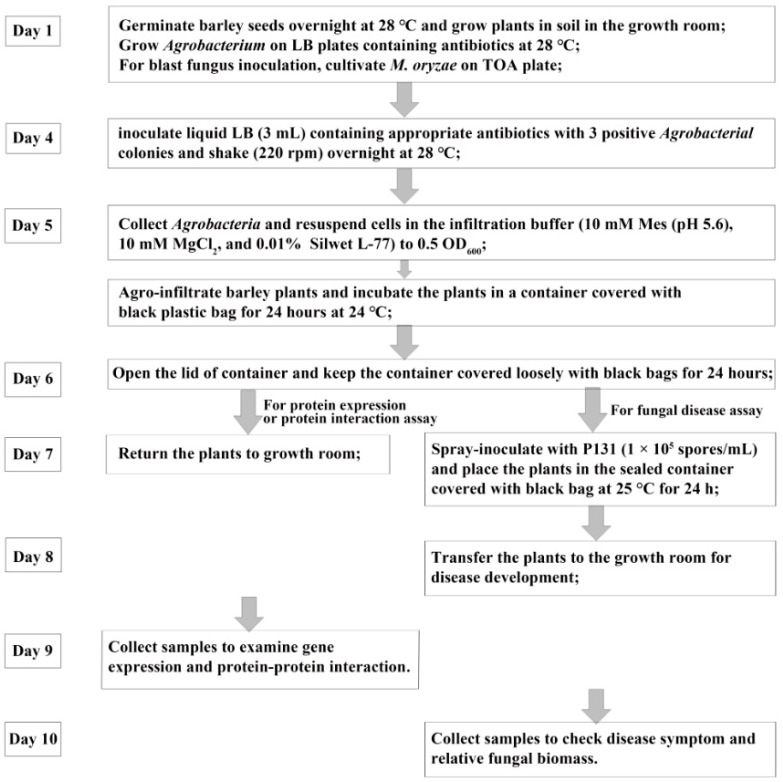
The flow chart of the AMTE. The details of experimental conditions can be found in Materials and Methods.

The above investigations helped us establish a rapid and highly efficient transient expression procedure for gene function analysis in barley plants: infiltrate the first leaf of 4- to 5-d-old barley seedlings with AGL1 (OD_600_ 0.5) harboring pCBEP (with insertion of cDNAs from the gene of interest); remove the excessive solutions from the leaf surface; keep the infiltrated plants in moisturizing condition/darkness for 1 and 2 days, respectively; return the plants to normal growth conditions and culture them for an additional 2 days for a functional assay of gene expression (Figure 3).

### 2.3. Transient Expression with pCBEP:GUS in Other Monocot Plants

**Figure 4:**
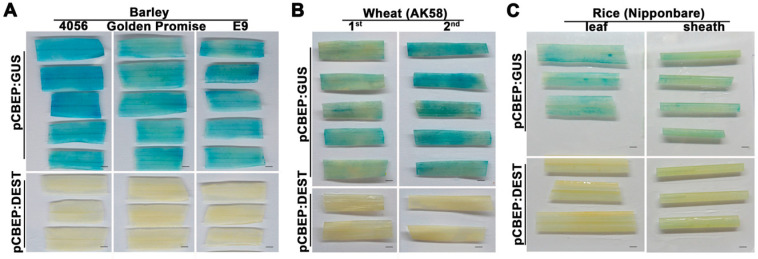
Transient expression with pCBEP:GUS in barley, wheat, and rice plants. (**A**). Expression of GUS in the first leaves of different barley varieties. E9 was used as control. (**B**). Expression of GUS in the 1st and 2nd leaves of wheat plants (AK58). (**C**). Expression of GUS in leaf and sheath of rice plants (Nipponbare). The plants were syringe-infiltrated (**A**,**B**) or vacuum-infiltrated (**C**) with 0.5 OD_600_ of AGL1 (pCBEP:GUS) following 1 d moisturizing/2 d darkness treatment; samples were collected at 4 dpi. Expression of empty vector (pCBEP:DEST) used as a negative control. Scale bars = 1.5 mm.

In order to test whether the transient expression procedure was adaptable to other monocots, we first examined the GUS expression in two different varieties (4056 and Golden Promise) of barley. The agro-infiltrated leaves were collected at 4 dpi, and GUS staining results showed that tissues of these two varieties were successfully stained as the E9 control (Figure 4A), indicating that the transient expression procedure is effective for different barley varieties. We further evaluated the transient expression of GUS in wheat and rice plants. The first and second leaves of 7-day-old wheat seedlings were syringe-infiltrated, and the leaf and sheath of 2-week-old rice plants were vacuum-infiltrated with the AGL1 (pCBEP:GUS). The results showed that GUS staining in the second leaf of wheat plants was more robust than that in the first leaf and was comparable to the levels in barley leaves (Figure 4B). However, the staining of rice leaf and sheath tissues was less intense (Figure 4C), implying an attenuated expression of GUS in rice plants.

### 2.4. AGL1 (pCBEP)-Mediated Transient Expression for Analyzing Protein-Protein Interactions in Monocots

**Figure 5:**
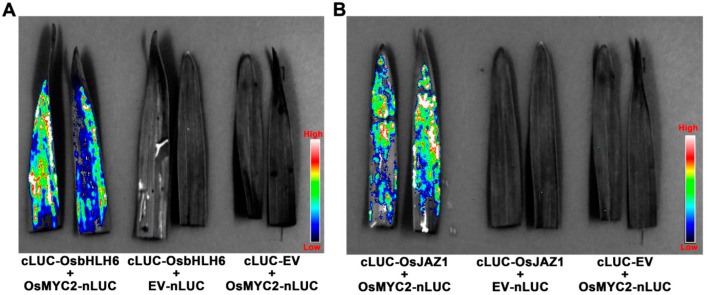
The optimized AMTE was amenable to Split-LUC assay of protein-protein interaction in barley (var. E9). The cLUC-OsbHLH6 and OsMYC2-Nluc (**A**) and the cLUC-OsJAZ1 and OsMYC2-Nluc (**B**) were transiently expressed in barley leaves. Samples were assayed at 4 dpi. Co-expressions with cLUC-EV or EV-nLUC were used as the control.

AMTE is widely used for the characterization of gene function in plants; however, monocots are rarely used due to the low levels of proteins expressed in plant cells. To investigate whether AGL1 (pCBEP)-mediated transient expression could produce adequate levels of proteins suitable for functional assays in barley plants, we applied the procedure to the split-luciferase (Split-LUC) assay, widely used for analyzing protein-protein interactions. Since interactions between rice OsMYC2 and OsbHLH6 or OsJAZ1 have been demonstrated in *N. benthamiana* [28], we fused the coding sequences of these proteins with those of luciferase and constructed pCBEP vectors expressing the fusion proteins. When barley leaves were co-infiltrated with AGL1 strains carrying the pCBEP constructs, prominent light signals could be observed in leaves where OsMYC2-nLUC and cLUC-OsbHLH6 or cLUC-OsJAZ1 and OsMYC2-nLUC were co-expressed; no luciferase signals could be observed in leaves where these proteins were co-expressed with cLUC-EV or EV-nLUC (Figure 5). These results demonstrated that OsMYC2 interacted with OsbHLH6 and OsJAZ1, indicating that AGL1 (pCBEP)-mediated transient expression can accurately detect protein–protein interactions in intact seedlings of monocots.

### 2.5. Functional Screening with AGL1 (pCBEP)-Mediated Transient Expression for Rice Genes Conferring Disease Susceptibility

We have constructed a full-length cDNA library from transcripts upregulated in the compatible interaction between rice and blast fungus, and several cDNA clones have been identified as capable of inducing cell death when transiently expressed with a 35S promoter on *N. benthamiana* [5]. To directly screen for genes promoting blast disease, we introduced the full-length cDNA library into the binary vector pCBEP-DEST to construct the expression library through Gateway^®^ cloning. We also examined the impact of agro-infiltration on the interaction between barley and blast fungus. The results showed that the pathogen successfully caused disease on agro-infiltrated leaves, although the disease symptoms and lesion areas were slightly reduced (Figure S3). Subsequently, the *Agrobacterium* strain AGL1 was transformed with the expression library, and the resulting individual transformants were used for transient expression in barley leaves, which were further challenged with *M. oryzae* P131 (Figure 3). After three rounds of screening, 15 clones were found, from about 2000 individual clones, to enhance lesion formation compared with the GUS control (Table 1). The results indicated that these candidate genes might play important roles in the barley-*M. oryzae* interaction.

A sequence analysis revealed that these candidate genes encode rice proteins with diverse annotated functions, among which three were transcription factors with NAC or zinc finger domains; four were chloroplast-related proteins, including nudix hydrolase, pheophytinase, adenylate kinase, and magnesium transporter; seven were proteins with other functions, such as cytochrome and its oxidase, cysteine proteinase inhibitor, purine permease, PH-domain protein, CTL-like protein, and ribosomal protein. However, there was one protein with an unknown function (Table 1).

### 2.6. Overexpression of the Chloroplast-Related Proteins Promoted Disease Susceptibility in Barley and Arabidopsis

**Figure 6:**
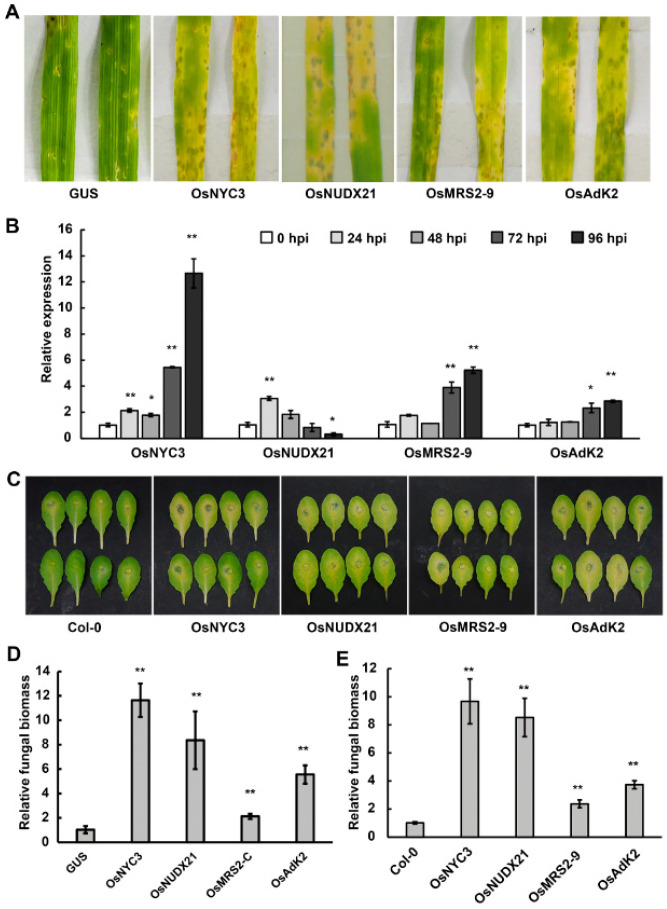
Functional analysis of the chloroplast-related genes *OsNYC3*, *OsNUDX21*, *OsMRS2–9*, and *OsAk2* on barley and *Arabidopsis*. (**A**). Transient expression of candidate genes enhanced blast disease symptoms on barley leaves. At 2 d after agro-infiltration, barley leaves were spray-inoculated with blast fungus P131 (1 × 10^5^ spores/mL) and incubated in a dark growth chamber at 25 °C for 24 h, followed by a 16-h light/8-h darkness photoperiod. Photographs were taken 3 days after fungal inoculation. (**B**). Relative transcript levels of candidate rice genes during rice blast disease. Quantitative RT-PCR data were normalized against the rice housekeeping gene *ACTIN*. Data shown are means ± SD (*n* = 3). Asterisks indicate significant differences calculated by Student’s *t*-test (* *p* < 0.05, ** *p* < 0.01). Three biological replicates were performed. (**C**). Overexpression of candidate genes in transgenic *Arabidopsis* lines promoted disease caused by *Colletotrichum higginsianum*. Detached leaves of 5-week-old plants were spot-inoculated with Ch-1 (1 × 10^5^ spores/mL) and incubated in a plastic box at 25 °C in darkness. Photos were taken 5 days post-inoculation. The above experiments were repeated three times, and similar results were observed. (**D**,**E**). Relative fungal biomass in inoculated barley (**D**) and *Arabidopsis* (**E**) leaves. The relative fungal biomass in samples collected in (**A**,**C**) was determined by DNA-based qPCR. Levels of the *M. oryzae actin* gene were normalized against the barley *actin* gene, and levels of *C. higginsianum β-Tublin* were normalized against the *Arabidopsis actin* gene. A student’s *t*-test was used to test significance and generate *p*-values (** *p* < 0.01).

Chloroplasts play a central role in the regulation of plant resistance to pathogens [29,30]. Several chloroplast-related proteins have been reported to manipulate defense hormones, ROS, or secondary metabolism to increase disease susceptibility [31,32,33,34]. To confirm the role of the identified chloroplast-related genes in disease susceptibility, we amplified the full length of the coding regions of the *OsNYC3* (pheophytinase in α/β hydrolase-fold family), *OsNUDX21* (nudix hydrolase), *OsMRS2–9* (magnesium transporter), and *OsAk2* (adenylate kinase) genes and cloned them into the binary vector pCBEP-DEST. Transient overexpression of these genes on barley strongly enhanced the disease symptoms and relative biomasses of the blast fungus (Figure 6A,D). In addition, a qRT-PCR assay revealed that transcript levels of these chloroplast-related genes were significantly upregulated during the P131 infection of rice plants (Figure 6B), suggesting that they are involved in the interaction between rice and blast fungus.

In order to rule out the potential impact of *Agrobacteria* on the transient assay of disease susceptibility on barley plants, we generated *Arabidopsis* transgenic lines constitutively overexpressing individual *OsNYC3*, *OsNUDX21*, *OsMRS2–9*, and *OsAk2* genes. The resulting transgenic lines were challenged with the hemibiotrophic pathogen *Colletotrichum higginsianum* strain Ch-1. At 5 days after inoculation, leaves from transgenic lines displayed enhanced symptoms with larger areas of chlorosis and lesions compared with the control lines overexpressing the *GUS* gene (Figure 6C). Accordingly, qRT-PCR assays showed that the overexpression lines accommodated more fungal biomass than wild-type Col-0 (Figure 6E), indicating that the upregulation of *OsNYC3*, *OsNUDX21*, *OsMRS2–9*, and *OsAk2* genes enhanced plant susceptibility to *C. higginsianum*.

## 3. Discussion

This study developed an economical, efficient, and reproducible AMTE method for gene function assays in monocots. The binary vectors initially used in this study, except for pCBEP, have been widely used for stable overexpression of target genes in monocots, yet we found dramatic variation in the efficiency of transient expression of GUS on barley but not on *N. benthamiana* plants; GUS activity was undetectable for AGL1 carrying vectors pINDEX and pTA7002 (Figure 1), indicating that they are incompatible with the transient expression on barley. Moreover, pCBEP, the vector giving the highest GUS expression on barley, was derived from pER8 but lacked the gene encoding synthetic XVE transcription factor capable of binding to the LexA-35S minimal promoter [25]. Vector pCBEP was originally designed for chemically-inducible gene expression in *Arabidopsis* plants bearing the pER8 T-DNA; the expression of pCBEP:GUS in stable transgenic lines was tightly controlled by the estradiol treatment, which activates the XVE. Interestingly, in contrast to stable transformation, the XVE was dispensable for transient expression of pCBEP:GUS on both barley and *N. benthamiana* plants; furthermore, the GUS activity was attenuated on barley plants when the XVE-expressing AGL1(pER8) was co-infiltrated (Figure 1). These observations imply that the regulation of transient gene expression is distinct from stable expression, and further study on the underlying mechanism may help improve the efficiency of AMTE on monocots. 

Various factors, such as *Agrobacterium* strains, plant species or genotypes, and co-cultural conditions, have been reported to affect AMTE efficiency [27,35]. The optimization of these factors has been the general focus for further improvement of gene expression. Here, we did not find substantial variation in the compatibility of *Agrobacterium* strains with barley plants, as the difference in GUS activity across the tested strains was within a two-fold difference (Figure S2A); similarly, GUS levels in different barley varieties or even in wheat plants are comparable (Figure 4A,B), indicating that the AGL1 (pCBEP) may be broadly amenable to efficient transient expression in these species. However, the GUS expression was less intense in rice plants (Figure 4C), indicating reduced compatibility between rice and the AGL1 strain. Since AGL1 is not widely used for *Agrobacterium*-mediated transformation in rice, other more compatible strains such as EHA105 may be helpful to improve the expression efficiency of this species further [26]. Similarly, it has been reported that younger rice seedlings (less than a week old) and co-cultivation with *Agrobacteria* at a low temperature (20 °C) can also enhance the efficiency of transient expression [26]. These observations indicate that our AMTE procedure may be further improved for rice plants. We found that in addition to younger leaves, high humidity and darkness treatment of plants after agro-infiltration markedly increased the transformation efficiency (Figure 2A,B,D). Similar effects have been reported in other plant transient expression systems. For example, higher humidity during the co-cultural step increases the transformation efficiency of *Marchantia polymorpha* [36]; darkness treatment before or after agro-infiltration is common in transient expression in *Arabidopsis* [3,37]. These treatments may exert multiple physiological impacts on plant-*Agrobacterium* interaction, such as reducing epicuticular wax content [38], promoting growth and activation of *Agrobacterium* [36,39], and inhibiting ROS production, thereby increasing plant susceptibility [40].

The rice blast is one of the most devastating diseases threatening rice production [41], but the knowledge of mechanisms underlying plant disease susceptibility is very limited. As the blast fungus can infect crops such as wheat and barley in addition to rice, barley was often used as a susceptible host for pathogenicity assays of *M. oryzae* [42]. Currently, the AMTE *in planta* has not been widely used on monocots such as rice and barley, especially in the dissection of plant-microbe interactions. Here, by using the AMTE *in planta* assay, we identified 15 candidate genes promoting the blast disease on barley, indicating that they may play important roles in the barley-*M. oryzae* interaction (Table 1 and Figure 5B). Among these, the gene encoding C2H2 type zinc finger protein 36-like has been reported to regulate H_2_O_2_ degradation and confer plant susceptibility to blast fungus infection [43]; the genes encoding OsNAC92, OsNYC3, and OsCYP94C1 are also upregulated during the compatible interaction [44]. Studies on the *Arabidopsis* ortholog of OsCYP94C1 have revealed that AtCYP94C1, which shares over 55% of its sequence identity with OsCYP94C1, attenuates defense responses to *Botrytis cinerea* infection by changing the hormone oxidation ratio of JA-Ile [45,46]. For most of the 15 candidate genes, further experiments are necessary to elucidate how they promote disease susceptibility in the host plants.

Interestingly, barley and *Arabidopsis* plants overexpressing the four chloroplast-related proteins exhibited intense symptoms of leaf chlorosis, or yellowing, the most prominent feature of senescence, following pathogen infection (Figure 6A,C). It is well known that biotic stresses could induce leaf senescence [47,48]; however, the underlying regulatory mechanism connecting senescence and disease susceptibility is not well understood. The OsNYC3 gene has been reported to regulate chlorophyll degradation, and the gene is highly expressed in dark-induced senescent leaves [49]. Similarly, other pheophytinases, the orthologs in *Arabidopsis* [50] and *Zoysia japonica* [51], accelerate chlorophyll degradation to promote leaf senescence. Moreover, senescent plants accumulate ABA and soluble sugar contents [51], which may benefit pathogen growth and promote disease susceptibility [52]. Thus, the blast fungus may upregulate the expression of rice pheophytinases OsNYC3 to hijack the senescence program to boost disease. Many chloroplast-related proteins are involved in other processes, such as amino acid synthesis and secondary metabolism, to negatively regulate plant immunity against pathogens [34,53]. For instance, many family members of nudix hydrolases, homologs of the OsNUDX21 identified in this study, control the production of a variety of metabolites and participate in a wide range of physiological processes [54,55]. Particularly, *Arabidopsis* NUDT7 [31] and wheat TaNUDX23 [33,56] have been identified to negatively regulate EDS1-dependent SA biosynthesis and ROS accumulation to facilitate pathogen infection. Further investigation is needed to elucidate whether OsNUDX21 functions to suppress the SA pathway to support the blast disease. In contrast to *OsNYC3* and *OsNUDX21*, which were induced 24 h after fungal infection, transcript levels of *OsMRS2–9* and *OsAK2* were upregulated at 72 hpi (Figure 6B), indicating these two genes might be associated more closely with the necrotrophic stage of pathogenesis. However, the molecular basis underlying the relationship between disease susceptibility and OsMRS2–9 or OsAK2 is unknown. Since *OsMRS2–9* and *OsAK2* belong to families with 9 and 7 members [57,58], respectively, and the effect of overexpression of *OsMRS2–9* and *OsAK2* on fungal biomass was weaker than that of *OsNYC3* and *OsNUDX21* in *Arabidopsis* (Figure 6E), it would be interesting to survey the expression profiles of other family members to find additional players in the compatible interaction. Nevertheless, the loss-of-function assay of these candidate genes is essential to pin down their role in plant disease susceptibility.

Furthermore, blast fungus is a typical hemibiotrophic pathogen, which establishes biotrophic growth in the early stage of infection and switches to necrotrophic growth afterwards. The infection process of this pathogen is highly dynamic and orchestrated by massive transcriptome reprograming in both the pathogen and the host plant [59,60]. Transcriptional profiling has been used to understand the molecular basis underlying the interaction; however, the roles of many specific genes in disease susceptibility are still largely unknown. The AMTE procedure established in this study efficiently assigned the function of specific genes in the promotion of disease processes. Notably, the 15 genes identified from nearly 2000 clones are distinct, indicating that the screening is not saturated and that further work may reveal more genes underpinning disease susceptibility. Moreover, most of the genes identified in this work are members of gene families, which highlights the potential existence of gene function redundancy in disease susceptibility. Functions of such genes are normally difficult to reveal by loss-of-function assays of single genes [61,62]. Thus, AMTE-based gain-of-function strategies provide a valuable toolkit for addressing questions of gene redundancy. We believe, with some modifications, the AMTE procedure can also be applied to *in planta* assays of gene function in disease resistance or abiotic stresses in monocot plants.

## 4. Materials and Methods

### 4.1. Plant Materials and Growth Conditions

The barley cultivars (*Hordeum vulgare* cv. 4056, Golden Promise, and E9), wheat cultivar (*Triticum aestivum* cv. AK58), rice cultivars (*Oryza sativa* cv. LTH and Nipponbare), and *Arabidopsis thaliana* Col-0 were used for this study.

The soil for rice growth was mixed with compost (BAIHUAHUI, Fuyu, China) and vermiculite (1:1); the soil for barley, wheat, *N. benthamiana*, and *Arabidopsis* was mixed with compost (PINDSTRUP, Pindstrup, Denmark) and vermiculite (1:1). Pots (6 cm) were filled with the mixed soil, keeping it consistently moist for sowing.

In pre-germination, rice seeds were soaked in water overnight, then surface disinfected with 3% NaClO for 10 min, rinsed repeatedly at least three times with sterilized water, and kept at 28 °C for 2 d in dark and wet conditions; the seeds of barley and wheat were soaked in water overnight, then kept at 28 °C for 1 d in dark and wet conditions; the seeds of *Arabidopsis* were suspended in 0.07% agarose solution at 4 °C for 3 d. *N. benthamiana* seeds were planted directly on the soil surface and covered with a transparent lid to moisturize for 2–3 days.

Barley, wheat, and *N. benthamiana* were grown at 24 °C in a growth room with a 16-h light/8-h dark cycle. One-leaf-stage barley, two-leaf-stage wheat, and 5-week-old *N. benthamiana* plants were used for *Agrobacteria*-mediated transient expression. Two-leaf-stage rice seedlings were grown at 28 °C in a growth room with a 16-h light/8-h dark cycle; rice cultivars LTH and Nipponbare were used for inoculation with blast fungus and *Agrobacteria*-mediated transient expression, respectively. *Arabidopsis thaliana* Col-0 and the transgenic lines were grown at 23 °C under a 10-h-light/14-h-dark cycle in the growth room, and 5-week-old seedlings were used for fungal infection. In each experiment, at least 16 plants were used for each treatment.

### 4.2. Vector Construction and Primers

Plasmids and primers used in this study are listed in Tables S1 and S2, respectively.

Additionally, to construct the vectors used for transient expression of GUS *in planta*, primers with *attB* sites were used to amplify the GUS coding sequence with M5 Magic Neo High-Fidelity DNA Polymerase (Mei5bio, Beijing, China) as described [5]. The amplified products were recombined into pDONR207 to generate an entry clone via gateway cloning (Thermo Fisher Scientific, Waltham, MA, USA) according to the manufacturer’s instructions. The coding sequence of 3 × Flag was added by PCR to the 3′ end of the coding region of GUS on the entry vector. The resulting entry vectors were recombined with the binary vectors in Table S1 to generate binary vectors expressing GUS. To delete the right border of the binary expression vector, pCBEP:GUS was used as the temple for amplification; the amplified product was treated with T4 polynucleotide kinase (Takara Bio, Dalian, China) and self-ligated with T4 DNA ligase (Takara Bio, Dalian, China) according to the manufacturer’s instructions.

To construct vectors for the Split-LUC assay, the amplification products of *attB1-cLUC-SfiI-attB2* or *attB1-SfiI-nLUC-attB2* were recombined into pDONR207 and subsequently recombined into the binary vector pCBEP:DEST via Gateway cloning. The sequences of *OsbHLH6* (Os04g0301500), *OsJAZ1* (Os04g0653000), and *OsMYC2* (Os10g0575000) were amplified from cDNA of rice (Nipponbare) and cloned into the *SfiI* sites of pCBEP:cLUC and pCBEP:nLUC, respectively, to obtain pCBEP:cLUC-OsbHLH6, pCBEP:cLUC-OsJAZ1, and pCBEP:OsMYC2-nLUC, as described previously [63].

To construct vectors for transient expression in barley and stable overexpression in *Arabidopsis* of the candidate genes, the coding sequences of *OsNYC3*, *OsNUDX21*, *OsMRS2–9*, and *OsAk2* were amplified from cDNA of rice (Nipponbare) and cloned into the binary vectors pCBEP:FLAG and pCBCS [63].

### 4.3. Agrobacterium Preparations

The binary vectors used for transient expression were transformed into the *Agrobacterium* strains AGL1, GV3101, EHA105, and C58C1 via electroporation [2]. A single positive colony of fresh *Agrobacteria* was shaken (220 rpm) overnight in liquid LB medium with rifampicin and appropriate antibiotics (Table S1) at 28 °C. After centrifugation (12,000 rpm, 5 min) and washing (adding 5 mL sterile ddH_2_O then re-centrifugation), bacterial pellets were resuspended in the infiltration buffer (10 mM Mes (pH 5.6), 10 mM MgCl_2_, and 0.01% Silwet L-77) to 0.5 OD_600_. For the acetosyringone (AS) treatment, 400 μM of AS was added to the infiltration buffer. 

### 4.4. Agrobacterium-Mediated Transient Transformation

The *Agrobacterium*-mediated transient transformation was performed according to previous reports [23], with a slight modification. The *Agrobacterium* suspension was infiltrated into the adaxial side of barley leaves with a 1 mL needleless syringe. After removing the excessive solution from the leaf surface, the infiltrated seedlings were placed into a sealed plastic container to keep the relative humidity above 98% (for 0–4 days). For dark treatments (0–2 d), black plastic bags were used to cover the containers with the seedlings after infiltration. Then the transformed plants were grown in the growth room (24 °C, 16-h light/8-h dark). For the treatment with inducers, 100 μM estradiol or 20 μM DEX was infiltrated 2 h after bacterial infiltration [22,24].

For vacuum infiltration, seedlings were inverted and gently put into a glass tube (20 × 200 mm) containing 50 mL of the infiltration medium and covered with filter paper. The uncapped tubes were placed inside a desiccator and vacuumed for over 20 min until plant leaves were infiltrated. After vacuum infiltration, excessive solution on the seedlings was removed with tissue papers, and seedlings were replanted in the soil for further growth.

### 4.5. GUS Assays

The histochemical assay was performed as previously described by Burman et al. [26]. In addition, *Agrobacteria*-infiltrated leaves were cut into sections of roughly equal size and immersed in GUS staining buffer (50 mM NaHPO_4_/NaH_2_PO_4_ (pH 7.0), 2 mM K_3_Fe(CN)_6_, 2 mM K_4_Fe(CN)_6_, 0.1% Triton X-100, and 1 mM X-Gluc), followed by vacuuming for 15 min. The staining was placed in the dark at 37 °C (barley for 12 h, *N. benthamiana* for 4 h). The sections were rinsed in 70% ethanol and washed three times in 95% ethanol until the chlorophyll was removed.

A quantitative fluorescent β-galactosidase assay of GUS activity was conducted as follows: Barley seedlings were infiltrated with *Agrobacteria* harboring individual binary vectors. The infiltrated tissues were ground in liquid nitrogen, and 50 mg of powder was resuspended and ground with 500 μL of extraction buffer (50 mM NaHPO_4_/NaH_2_PO_4_ (pH 7.0), 10 mM EDTA (pH 8.0), 0.1% Triton X-100, and 10 mM β-mercaptoethanol) on ice [64]. The extracts were subjected to centrifugation (12,000 rpm, 4 °C, 15 min), and 4 μL of the supernatant was transferred into 96-well microtiter plates for protein quantification by Bradford assay [65]. Meanwhile, 5 μL of the supernatant was mixed in 45 μL MUG buffer (20 mM MUG: methanol: extraction buffer = 1:4:23) for fluorescent reactions in the dark at 37 °C for 30 min, and 5 μL of the reaction products at 0 min and 30 min were separately transferred into 200 μL stop buffer (0.2 M Na_2_CO_3_) in 96-well flat bottom black polystyrene plates to stop the enzyme reaction. The fluorimeter of each plate was calibrated with 4-MU standards (5 nM 4-MU was added to a 200 μL stop buffer, and then 1:2 serial dilutions were prepared with the stop buffer to obtain the equation for the calibration curve). Fluorescence was measured with excitation at 365 nm and emission at 485 nm on a Tecan F200 spectrofluorimeter, with the number of flashes set at 20 nm [64]. Each sample has six technical repeats. The experiments were repeated three times.

### 4.6. Western Blot Assay of GUS Protein

The western blot assay on barley leaves was conducted according to previous reports [2,63]. To detect the GUS protein accumulation, 50 mg leaf samples were collected in 1.5 mL Eppendorf tubes containing two steel beads and frozen with liquid nitrogen before being ground into powder using a tissue lyser (JXFSTPRP-24L, Shanghai, China) at 45 Hz for 60 s. The tissue powder was resuspended in 100 μL loading buffer (8 M urea, 2% SDS, 20% glycerol, 100 mM Tris-HCl (pH 6.8), and 0.004% bromophenol blue), then boiled for 10 min and centrifuged at 12,000 rpm for 10 min. An aliquot of 20 μL of the resulting supernatant was resolved with 10% SDS-PAGE, followed by transferring protein from the gel onto a Hybond-P membrane by electroblotting (300 mA, 1.5 h) and subsequent immunoblot assays with an anti-FLAG (A8592; Sigma-Aldrich, Louis, MO, USA) antibody. Equal loading of samples was confirmed by Ponceau staining, as previously described [66].

### 4.7. Split-Luciferase Assay

The Split-LUC assay on barley leaves was conducted according to previous reports [67]. The *Agrobacterium* suspension pair with a final concentration of 0.5 OD_600_ was used for transient expression on barley. The co-infiltrated barley leaves at 4 dpi were sprayed with 100 μM luciferin (Promega, Madison, WI, USA) and kept in the dark for 5 min. Luminescence images were taken with a charge-coupled device (CCD) imaging system (Tanon, 5200 Multi, Shanghai, China).

### 4.8. Transformation of Arabidopsis Plants

*Arabidopsis* Col-0 plants were transformed by the floral dip method [68], with *A. tumefaciens* strain GV3101 harboring the indicated binary vectors (Table S1). *A. tumefaciens inoculum* was resuspended to 0.8 OD_600_ with buffer (0.04% Silwet L-77 (OSiSpecialties, Inc., Danbury, CT, USA) and 5% sucrose). The transgenic lines were screened by Basta (0.2% *v*/*v*, spraying twice for 1-week-old seedlings).

### 4.9. Plant Inoculations and Disease Assay

For the blast disease assay, conidia of *M. oryzae* strain P131 were prepared as described [69]. Two-leaf-stage rice seedlings or barley seedlings at 2 d after agro-infiltration were spray-inoculated with conidial suspensions (1 × 10^5^ conidia mL^−1^, each pot 3 mL). The inoculated rice and barley were incubated at 28 °C and 25 °C, respectively, in a sealed black plastic container for 24 h to maintain darkness and high relative humidity, followed by culturing in the growth room to allow further development of the disease [70]. For the *Arabidopsis* anthracnose disease assay, conidia of *C. higginsianum* strain Ch-1 were prepared as described [71]. Newly expanded leaves of 5-week-old *Arabidopsis* were detached and placed in a plastic container and spot-inoculated with 10 μL conidial suspensions (1 × 10^5^ conidia mL^−1^). The container was sealed and incubated in darkness at 25 °C. The plants or detached leaves were treated with sterile, deionized water as a control.

The disease severity of barley blast was classified based on the relative leaf lesion area calculated with the Image J software [72]. Genes with a relative lesion area higher than the pCBEP:GUS control (<40%) were selected as candidate susceptibility genes. The relative fungal biomass assay was used to further corroborate gene function in promoting disease.

### 4.10. Relative Fungal Biomass Assay

The genomic DNA of six infected leaves was extracted by 2% CTAB as described in [73], and the amount of fungal DNA was quantified with a qPCR assay [74]. The *MoActin* (MGG_03982) of the blast fungus was normalized against the barley reference gene *HvActin* (LOC123430406), and *β-tubulin* (CH63R_14125) of the *C. higginsianum* was normalized against the *Arabidopsis* reference gene *UBQ10* (AT4G05320). These genes were amplified by ChamQ Universal SYBR qPCR Master Mix (Vazyme, Nanjing, China) and a qPCR assay on an ABI QuantStudio 6 Flex (Thermo Fisher, Waltham, MA, USA). Each sample has three technical replicates. The experiments were repeated three times. Primer sequences are listed in Table S2.

### 4.11. Gene Expression Analysis

The total RNA was extracted from six inoculated rice leaves by SuperTRIgent (Mei5bio, Beijing, China), and cDNA was synthesized by the M5 Super Plus qPCR RT kit with gDNA remover (Mei5bio, Beijing, China) according to the manufacturer’s instructions. Transcript levels of candidate genes were determined by qPCR amplification and assay, as described above. The rice gene *OsActin* (*Os03g0718100*) was used as the reference gene. Each sample has three technical replicates. The experiments were repeated three times. The data were analyzed according to the 2^−ΔΔCT^ method [75]. The primers used in this study are listed in Table S2.

### 4.12. Statistical Data Analysis

The data were plotted with GraphPad Prism 8.0 (Graphpad, San Diego, CA, USA), and statistical analyses were performed using an unpaired *t*-test and a one-way ANOVA using SPSS 20.0 software (IBM, Amunk, NY, USA).

## Figures and Tables

**Table 1:** Information on the candidate genes enhancing susceptibility.

Clone No.	Gene	Encoding Protein	Disease Severity ^a^
7B210	*Os06g0354700*	Pheophytinase, NYC3	+++
27A6	*Os08g0109300*	Adenylate monophosphate kinase 2, AdK2	++
15A5	*Os02g0734300*	Nudix hydrolase 21, NUDX21	++
15C1	*Os04g0501100*	Magnesium transporter MRS2-C	++
357	*Os12g0150200*	Cytochrome P450, CYP94C1	++
367	*Os03g0327100*	NAC transcription factor 39, OsNAC92	++
5C8	*Os04g0498200*	Cytochrome c oxidase subunit 6b-2,COX6b2	++
32B1	*Os03g0437200*	C2H2 type zinc finger protein 36-like, ZFP36 (Bsr-d1)	+
1D8	*Os03g0145600*	CCCH type zinc finger protein 48	+
4F7	*Os05g0494200*	Cysteine proteinase inhibitor 2-like	+
6B3	*Os04g0587400*	Purine permease 11	+
9C7	*Os01g0856800*	Pleckstrin homology (PH) domain protein 1	+
9C3	*Os07g0656100*	C-type lectin (CTL)-like protein	+
2B21	*Os06g0181566*	60S ribosomal protein L39	+
4A46	*Os01g0562600*	Uncharacterized protein	+

^a^ Transient expression of candidate clones was performed as described. The disease severity was measured based on the barley leaf lesion area caused by the blast fungus infection. “+++”, “++”, and “+” indicate the lesion area is 80–100%, 60–80%, and 40–60% of the total leaf area, respectively; the pCBEP:GUS was used as a control with lesion area < 40%.

## Data Availability

Not applicable.

## Supplementary Materials

The following supporting information can be downloaded at: https://www.mdpi.com/article/10.3390/ijms24087636/s1.
