# Supplementary material for: An Agrobacterium-Mediated Transient Expression Method for Functional Assay of Genes Promoting Disease in Monocots
Source: Int J Mol Sci. 2023 Apr 21;24(8):7636. doi: 10.3390/ijms24087636 (PMC10142106; doi:10.3390/ijms24087636)
Supplement: Supplementary file 1 [file ijms-24-07636-s001.zip › ijms-2289732-supplementary.pdf]

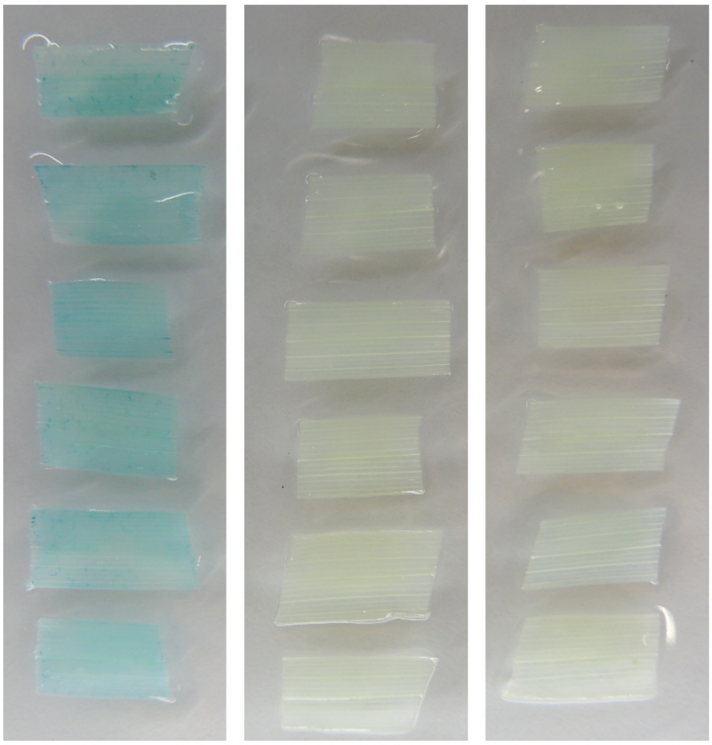

Figure S1 The GUS expression was dependent on the transfer of T-DNA. The right border sequences of pCBEP:GUS were deleted for transient expression. After Agro-infiltration, barley seedlings were incubated in growth room (24 °C, 16-h light/8-h darkness photoperiod) with 1 day of moisturizing treatment; samples were collected and analyzed at 4 dpi. The experiments were repeated three times, and similar results were observed.

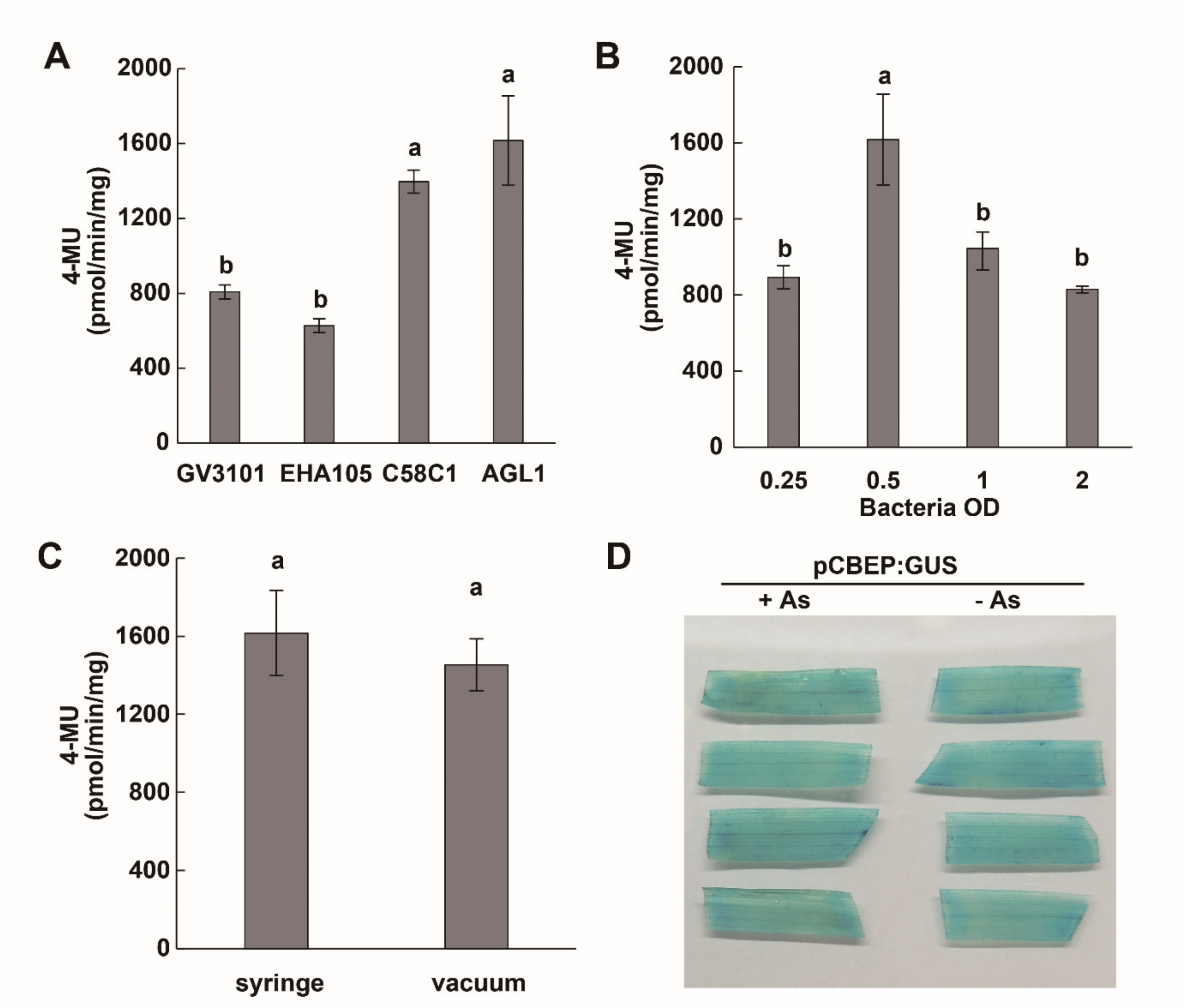

Figure S2 Optimization of transient expression in barley leaves. A. Quantification of GUS activity with diverse *Agrobacterium* strains (AGL1, C58C1, GV3101, and EHA105) harboring pCBEP:GUS. **B**. Different concentration (0.25-2 OD_{600}) of AGL1. **C**. Agro-infiltration by syringe or vacuum. **D**. With or without acetosyringone (AS) in the infiltration buffer. AGL1 (0.5 OD_{600}) harboring pCBEP:GUS was used for Agro-infiltration in the first leaf of barley with 1 day of moisturizing treatment; samples were collected and analyzed at 4 dpi. Data shown were means ± SD (n = 6). Different letters indicate significant differences at $P<0.01$. The experiments were repeated three times, and similar results were observed.

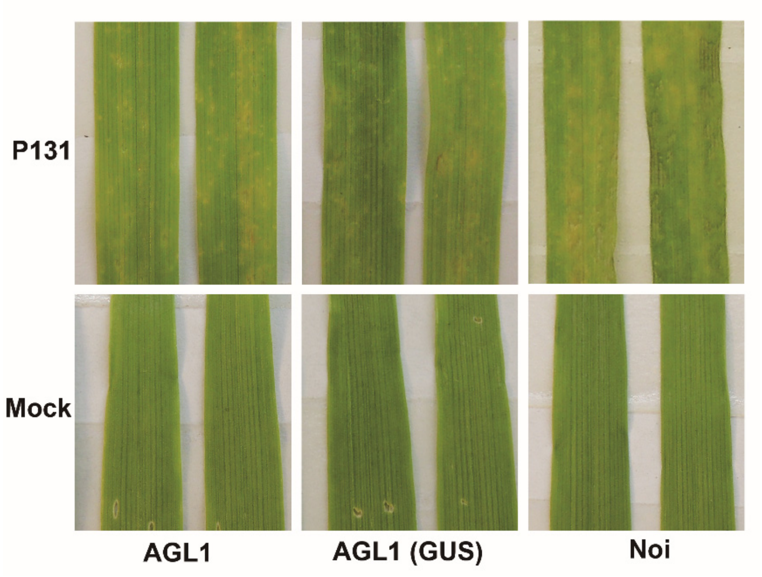

Figure S3 Blast fungus can successfully infect Agro-infiltrated leaves of barley. At 3 d after Agro-infiltration of the AGL1, AGL1 (GUS) or not (Noi), barley leaves were spray-inoculated with *M. oryzae* P131 (1×10^5 spores/mL) and incubated in a dark growth chamber at 25 °C for 24 h, followed by a 16-h light/8-h darkness photoperiod. The plants were sprayed with sterile deionized water as mock. Photographs were taken 3 days after fungal inoculation. The experiments were repeated three times, and similar results were observed.

Table S1. Plasmids used in this study

Plasmids	Relative properties or purpose	Source/References
pIPKb002	Constitutive binary expression vector, $Spec^R$	*Lu et al.,* 2016
pER8:DEST	Intermediate vector modified from pER8, inducible expression vector, $Spec^R$	Fang et al., 2017
pCBEP:DEST	Intermediate vector modified from pER8 to pCBER DEST, $Kana^R$	Fang et al., 2017
pINDEX2	Inducible expression vector, $Kana^R$	Ouwerkerk et al., 2001
pTA7002	Inducible expression vector, $Kana^R$	Aoyama and Chua, 1997
pCBDEST-transient	Constitutive expression vector, $Kana^R$	Chang et al., 2019
pIPKb002:GUS	Transient expression of *GUS*	This study
pER8:GUS	Transient expression of *GUS*	This study
pCBEP:GUS	Transient expression of *GUS*	This study
pCBEP:GUS-Flag	Transient expression of *GUS* tagged Flag	This study
pCBEP-noRB:GUS	Transient expression of *GUS* deleting the right border	This study
pINDEX2:GUS	Transient expression of *GUS*	This study
pTA7002:GUS	Transient expression of *GUS*	This study
pCBDEST-GUS	Transient expression of *GUS*	This study
pCBEP:cLUC	Transient expression of cLUC for Split-LUC assay	This study
pCBEP:nLUC	Transient expression of nLUC for Split-LUC assay	This study
pCBEP:cLUC-OsbHLH6	Transient expression of cLUC-OsbHLH6	This study
pCBEP:cLUC-OsJAZ1	Transient expression of cLUC-OsJAZ1	This study
pCBEP:OsMYC2-nLUC	Transient expression of OsMYC2-nLUC	This study
pCBEP:OsNYC3	Transient expression of OsNYC3	This study
pCBEP:OsNUDX21	Transient expression of OsNUDX21	This study
pCBEP:OsMRS2-9	Transient expression of OsMRS2-9	This study
pCBEP:OsAk2	Transient expression of OsAk2	This study
pCBCS	Constitutive binary expression vector, $Kana^R$	Chen et al., 2021
pCBCS:OsNYC3	Stable overexpression of OsNYC3 for *Arabidopsis transformation*	This study
pCBCS:OsNUDX21	Stable overexpression of OsNUDX21	This study
pCBCS:OsMRS2-9	Stable overexpression of OsMRS2-9	This study
pCBCS:OsAk2	Stable overexpression of OsAk2	This study

^{R}resistance: Spec, spectinomycin; Kana, kanamycin.

Table S2. Primers used in this study

Primers	Sequence 5'-3'	Purpose
GUS-attb-F	aaaaagcaggctATGTTACGTCCTGTAGAAACCCC	Cloning of *GUS*
GUS-attb-R	agaaagctgggtTTATTGTTTGCCTCCCTGCTG	
DEST-F	gcctcgacacgtgtaaGACTCTAGCCTCGAGAATCAACA	Cloning of the *attR* cassette
DEST-R	agatgtacatactcgaGATCGACTAGTATCAACCACTTTGT	
pCBEP noRB-F	TGGCGGCCGCTCTAGAACTA	Cloning of pCBEP:GUS without right border for self-ligated
pCBEP noRB-R	AATCCGATCCGCACATGAGG	
attb-c/nLUC-sfiI-F	aaaaagcaggctTACTATTCTAGTCGACCTGCAG	Cloning of cLUC and nLUC containing two different SfiI
attb-c/nLUC-sfiI-R	agaaagctgggtCTGGTGATTTTTGCGGACTCTA	sites to construct pCBEP:cLUC and pCBEP:nLUC
OsMYC2-sfiI-F	attaacaaggccattacggccATGTGGGTTTTGTTATCTCCTCTCCTC	Cloning of *OsMYC2* from cDNA of rice (Nipponbare) to
OsMYC2-sfiI-R	aactgattggccgaggcggccccCTCGGCGATGCGGGTGTAGA	construct pCBEP:OsMYC2-nLUC
OsbHLH6-sfiI-F	attaacaaggccattacggccATGGACGCCGAGATGGCCA	Cloning of *OsbHLH6* from cDNA of rice (Nipponbare) to
OsbHLH6-sfiI-R	aactgattggccgaggcggccccGCTCAACGGACTGCCGGT	construct pCBEP:cLUC-OsbHLH6
OsJAZ1-sfiI-F	attaacaaggccattacggccATGGATCTGTTGGAGAAGAA	Cloning of *OsJAZ1* from cDNA of rice (Nipponbare) to
OsJAZ1-sfiI-R	aactgattggccgaggcggccccCTGGGCCTTGCCCTCAGC	construct pCBEP:cLUC-OsJAZ1
attb-Flag-sfiI-F	aaaaagcaggctTACTATTCTAGTCGACCTGCAG	Cloning of Flag containing two different *SfiI* sites to
attb-Flag-sfiI-R	agaaagctgggtCTGGTGATTTTTGCGGACTCTA	construct pCBEP:Flag
SfiI-OsNYC3-F	attaacaaggccattacggccGCCACTAGTGTGGCCATGGAAGT	Cloning of *OsNYC3* from cDNA of rice (Nipponbare) to
SfiI-OsNYC3-R	aactgattggccgaggcggccccTCTAGATATTACCCATGTGTTG	construct pCBEP:OsNYC3 and pCBCS:OsNYC3
SfiI-OsNUDX21-F	attaacaaggccattacggccACAATCAAAGATTCGATGGCCG	Cloning of *OsNUDX21* from cDNA of rice (Nipponbare) to
SfiI-OsNUDX21-R	aactgattggccgaggcggccccCAGCGCAGACTGCAGCTTGTTG	construct pCBEP:OsNUDX21 and pCBCS:OsNUDX21
SfiI-OsMRS2-C-F	attaacaaggccattacggccGACCTCGACGGCCATGGACCAC	Cloning of *OsMRS2-C* from cDNA of rice (Nipponbare) to
SfiI-OsMRS2-C-R	aactgattggccgaggcggccccCAAGGGGAAAAACCTTCTCTTCTTG	construct pCBEP:OsMRS2-C and pCBCS:OsMRS2-C
SfiI-OsAdK2-F	attaacaaggccattacggccGTTAATATGGTGAAGGAACGCC	Cloning of *OsAdK2* from cDNA of rice (Nipponbare) to
SfiI-OsAdK2-R	aactgattggccgaggcggccccTGCGCTAGCCACCATTTCTGT	construct pCBEP:OsAdK2 and pCBCS:OsAdK2

MGActin-qF	GGTCTTGAGAGCGGTGGTATCCATG	*Magnaporthe oryzae* Actin gene for qPCR
MGActin-qR	TGCCAGGGCAGTGATCTCCTTC	
Barley actin F	CCTCGGCACACTGGTGTCATGGTA	Barley HvActin gene for qPCR
Barley actin R	AAGGGGCCTCAGTGAGCAACACA	
UBQ10-F	GGCCTTGTATAATCCCTGATGAAT	*Arabidopsis UBQ10* gene for qPCR
UBQ10-R	AAAGAGATAACAGGAACGGAAACA	
β-tubulin-F	CGGTCTCAAGATGTCCTCCAC	*Colletotrichum higginsianum β-tubulin* gene for qPCR
β-tubulin-R	AGAAAGCCTTGCGACGGAACA	
R-Actin1-F	TGGCATTGCTGACAGGATGAGC	Rice *OsActin* gene for qPCR
R-Actin1-R	CGTCGTACTCAGCCTTGGCAATC	
OsNYC3-qF	ATTACAACCATGGGCGGAGG	Rice *OsNYC3* gene for qPCR
OsNYC3-qR	TCCCAGAGAGTTCCCCACAA	
OsNUDX21-qF	CATGATGTTCCCCAAGGGCG	Rice *OsNUDX21* gene for qPCR
OsNUDX21-qR	TCTTGTAGTACCAGCAGCCG	
OsMRS2-C-qF	ACATTCGTGGTGGCCATCTT	Rice *OsMRS2-C* gene for qPCR
OsMRS2-C-qR	GTCCATTCGAACGCATGTGG	
OsAdK2-qF	AAGGTCATGATAGCAGGGGC	Rice *OsAdK2* gene for qPCR
OsAdK2-qR	AGCTCGCTTCCCATTCTCAC	
